# Demographic factors associated with response to topical Jak inhibitors in a diverse vitiligo cohort

**DOI:** 10.1016/j.xjidi.2026.100520

**Published:** 2026-08-03

**Authors:** Hira Rizwi, Britney T. Nguyen, Anastasia Chahine, Thabat Dahdoul, Jessica Shiu

**Affiliations:** 1 Department of Dermatology, University of California, Irvine, Irvine, California, USA; 2 Department of Medicine, University of California, Irvine, Irvine, California, USA; 3 ICTS BERD, University of California, Irvine, Irvine, California, USA

**Keywords:** Phototherapy, Public health, Vitiligo

## Abstract

Vitiligo is a chronic autoimmune disorder that causes progressive melanocyte destruction and a significant psychosocial burden. Topical Jak inhibitors including ruxolitinib cream, are a Food and Drug Administration–approved treatment option. Real-world data on treatment response across diverse patient populations remain limited. This single-institution retrospective cohort study (April 2018–2024) included 120 patients with vitiligo from a Southern California academic medical center. Eligibility criteria included ≥2 dermatology visits, Vitiligo Area Scoring Index scores, insurance documentation, and current topical Jak inhibitor treatment. Bivariate analyses and multivariable Firth logistic regression were performed. The cohort was 34.2% Asian, 16.6% Hispanic, and 49.2% White. On bivariate analyses, Hispanic ethnicity (50% vs 27% non-Hispanic, *P* = .042) and longer treatment duration (*P* = .010) were significantly associated with Vitiligo Area Scoring Index improvement. On multivariable analysis, treatment duration remained an independent predictor (adjusted OR = 1.11 per month, 95% confidence interval = 1.04–1.20, *P* < .001), whereas non-Hispanic ethnicity had 69% lower odds of improvement (adjusted OR = 0.307, 95% confidence interval = 0.099–0.912, *P* = .034). Publicly insured patients experienced higher Jak inhibitor denial rates (60% vs 40%, *P* = .018). In this diverse real-world cohort, Hispanic ethnicity and longer treatment duration were independently associated with Vitiligo Area Scoring Index improvement. Insurance-related disparities and limited representation of certain minority groups highlight inequities in access to vitiligo care. Larger studies that incorporate standardized outcomes are needed.

## Introduction

**Figure 1:**
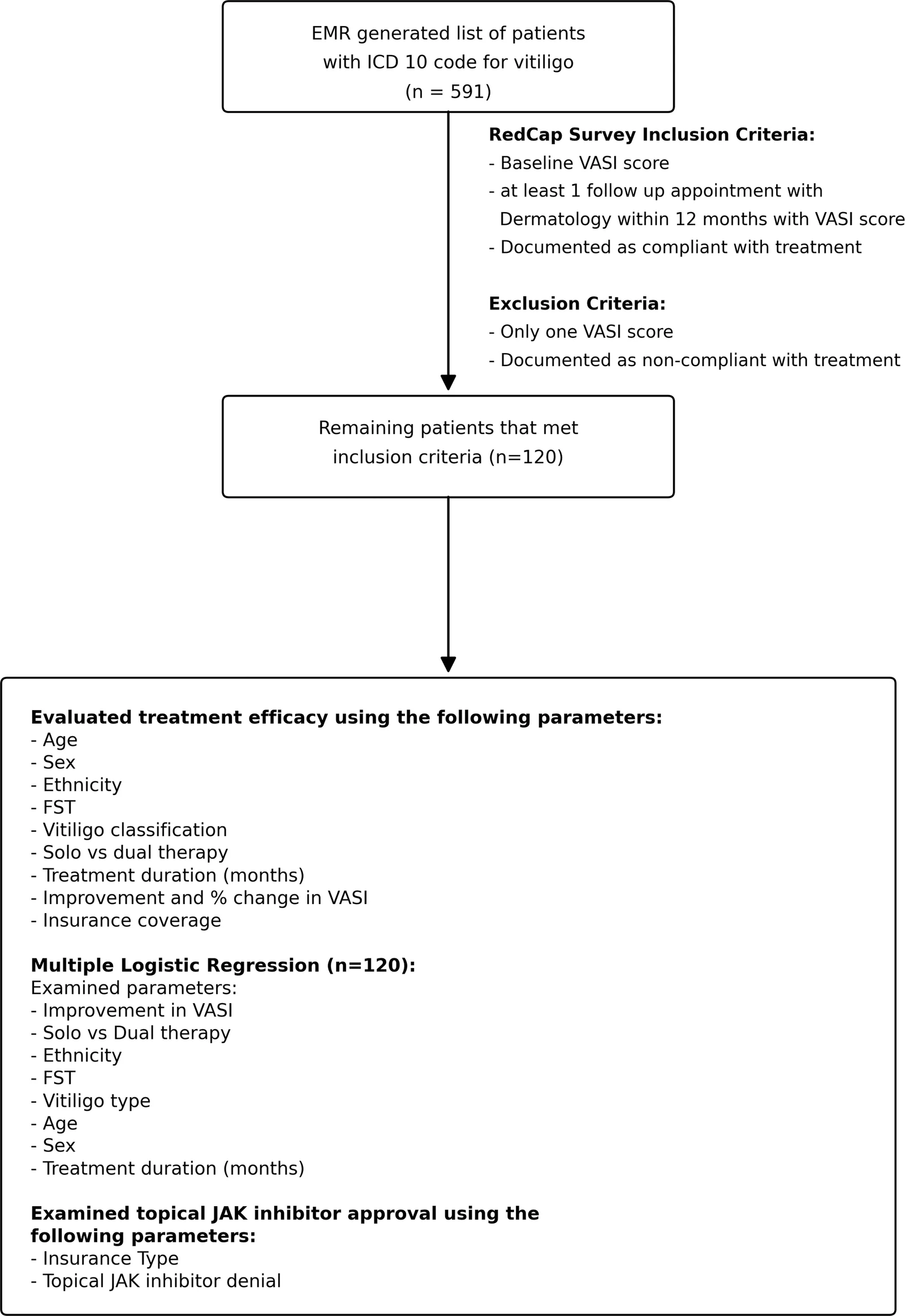
**Flow chart of patient population selection**.

Vitiligo is an autoimmune condition characterized by CD8^+^ T cell–mediated melanocyte destruction and causes significant psychosocial distress, especially in darker skin types (Bibeau et al, 2023). Conventional treatments, such as topical immunosuppressives and narrowband UVB, are time consuming and often yield an incomplete response (Nicolaidou et al, 2007). Ruxolitinib cream, a Jak1/2 inhibitor Food and Drug Administration approved for vitiligo in 2022, reduces chemokine release and autoimmune melanocyte destruction (Rosmarin et al, 2022). Other Jak inhibitors (Jakis), such as tofacitinib cream, also promote repigmentation. Combining topical Jakis with phototherapy enhances efficacy, but insurance denials and time constraints limit access (Thompson et al, 2020). Despite vitiligo’s prevalence among racial/ethnic minorities, over 80% of participants in the ruxolitinib phase 3 trials were White, leaving gaps in understanding how race/ethnicity impacts treatment response (Rosmarin et al, 2022). The Fitzpatrick Skin Type (FST), a scale used to characterize constitutive skin pigmentation and phototype, may also influence treatment response and the interpretation of repigmentation outcomes, yet remains understudied in topical Jaki trials. The Vitiligo Area Scoring Index (VASI) is a validated, clinician-administered instrument for quantifying disease extent and monitoring treatment response, ranging from 0 to 100, with lower scores indicating less depigmentation (Figure 1).

The objective of this study was to examine real-world treatment outcomes using a retrospective cohort study, with topical Jakis, with and without concurrent narrowband UVB phototherapy (dual therapy), in a diverse Southern California cohort at a single institution, using VASI scores to identify demographic and clinical predictors of treatment response, dual therapy use, and treatment approval. Patients with an International Classification of Disease, Tenth Revision diagnosis of vitiligo were identified through Epic’s SlicerDicer tool and included if they had ≥2 dermatology visits, recorded VASI scores, had insurance information, and were currently on topical Jaki treatment. Data were extracted through REDCap on 120 patients with vitiligo evaluated between April 2018 and 2024.

The cohort consisted of 34.2% Asian, 16.6% Hispanic, and 49.2% White patients. Vitiligo was classified according to the international consensus framework (Ezzedine et al, 2012) and distributed as follows: generalized (40%), localized (37.5%), acrofacial (18.3%), and segmental (4.2%), with a statistically significant nonuniform distribution (c^2^ *P* < .001) (Table 1). Hispanics had a higher prevalence of localized vitiligo (45%), whereas generalized vitiligo was more prevalent in Asians and White patients (Table 1). Bivariate analyses were conducted to identify variables individually associated with 3 outcomes of interest: VASI improvement, dual-therapy use, and topical Jaki treatment approval. Ethnicity was significantly associated with VASI improvement (*P* = .042) (Table 2). When improvement rates were calculated within each ethnic group, 50% of Hispanic patients (n = 10) improved, compared with 27% of non-Hispanic patients (n = 27) (Table 3). Longer treatment duration was also associated with VASI improvement (*P =* .010), whereas sex, FST, vitiligo classification, and dual therapy were not.

**Table 1:** Baseline Patient Characteristics (n = 120)

Characteristic	Value
Demographic characteristics	
Age (y), mean	42
Sex	
Male	57 (47.5%)
Female	63 (52.5%)
Ethnicity	
Asian	41 (34.2%)
Hispanic	20 (16.6%)
White	59 (49.2%)
Clinical characteristics	
Fitzpatrick skin type, mean	3
Treatment duration (mo), mean	9.67
Vitiligo classification^1^	
Generalized	48 (40%)
Asian	16 (39%)
Hispanic	5 (25%)
White	27 (45.8%)
Localized	45 (37.5%)
Asian	15 (36.6%)
Hispanic	9 (45%)
White	21 (35.6%)
Acrofacial	22 (18.3%)
Asian	6 (14.6%)
Hispanic	6 (30%)
White	10 (16.9%)
Segmental	5 (4.2%)
Asian	4 (9.8%)
Hispanic	0 (0%)
White	1 (1.7%)
Chi-square p-value	*P* < .001

1 Percentages for ethnicity-specific vitiligo subtype rows represent the proportion within each ethnic group (denominators: Asian = 41; Hispanic = 20; White = 59).

**Table 2:** Bivariate Analysis Table Comparing 3 Outcomes: (i) VASI improvement, (ii) Dual-Therapy Use, and (iii) Treatment Approval Status

Variable	No Improvement n = 83^1^	Improvement n = 37^1^	*P*-Value	No Dual Therapy n = 32^1^	Dual Therapy n = 88^1^	*P*-Value	Approval n = 110^3^	Denial n = 10^3^	*P*-Value^4^
Sex			.5			.083			
Male	41 (49%)	16 (43%)		11 (34%)	46 (52%)				
Female	42 (51%)	21 (57%)		21 (66%)	42 (48%)				
Ethnicity			.042			.14			
Hispanic	10 (12%)	10 (27%)		8 (25%)	12 (14%)				
Non-Hispanic	73 (88%)	27 (73%)		24 (75%)	76 (86%)				
Age (y)	41 ± 21 (med 42)	43 ± 20 (median 44)	.7	41 ± 19 (median 43)	43 ± 22 (median 43)	.7			
Fitzpatrick Skin Type			.7			.5			
1	3 (3.6%)	1 (2.7%)		0 (0%)	4 (4.5%)				
2	26 (31%)	9 (24%)		11 (34%)	24 (27%)				
3	29 (35%)	19 (51%)		13 (41%)	35 (40%)				
4	22 (27%)	7 (19%)		7 (22%)	22 (25%)				
5	2 (2.4%)	1 (2.7%)		0 (0%)	3 (3.4%)				
6	1 (1.2%)	0 (0%)		1 (3.1%)	0 (0%)				
Dual therapy			.7^2^						
No dual therapy	23 (28%)	9 (24%)							
Dual therapy	60 (72%)	28 (76%)							
Vitiligo classification			.2			.032			
Generalized	34 (41%)	14 (38%)		7 (22%)	41 (47%)				
Localized	34 (41%)	11 (30%)		16 (50%)	29 (33%)				
Acrofacial	11 (13%)	11 (30%)		6 (19%)	16 (18%)				
Segmental	4 (4.8%)	1 (2.7%)		3 (9.4%)	2 (2.3%)				
Treatment duration (mo)	8 ± 5 (med 7)	13 ± 10 (median 11)	.010	10 ± 10 (median 7)	10 ± 6 (median 10)	0.2			
VASI improvement									.7^2^
No improvement				23 (72%)	60 (68%)				
Improvement				9 (28%)	28 (32%)				
Insurance type									.018
Private							85 (77%)	4 (40%)	
Public							25 (23%)	6 (60%)	

Abbreviation: VASI, Vitiligo Area Scoring Index.

1 n (%); mean ± SD (median).

2 Pearson’s chi-square test, Wilcoxon rank-sum test, and Fisher’s exact test.

3 n (%).

4 Fisher’s exact test.

**Table 3:** Ethnicity-Stratified Clinical and Treatment Characteristics

Variable	Asian	Hispanic	White
Sex by ethnicity			
Male	18 (43.9%)	9 (45.0%)	30 (50.8%)
Female	23 (56.1%)	11 (55.0%)	29 (49.2%)
Insurance type by ethnicity			
Public	11 (26.8%)	8 (40.0%)	12 (20.3%)
Private	30 (73.2%)	12 (60.0%)	47 (79.7%)
Dual therapy by ethnicity			
Yes	31 (75.6%)	12 (60.0%)	45 (76.3%)
No	10 (24.4%)	8 (40.0%)	14 (23.7%)
Topical Jaki decision by ethnicity			
Approval	39 (95.1%)	18 (90.0%)	53 (89.8%)
Denial	2 (4.9%)	2 (10.0%)	6 (10.2%)
VASI improvement by ethnicity			
Improved	17 (41.5%)	10 (50.0%)	10 (16.9%)
Not Improved	24 (58.5%)	10 (50.0%)	49 (83.1%)

Abbreviations: Jaki, Jak inhibitor; VASI, Vitiligo Area Scoring Index.

Dual therapy, defined as concurrent topical Jaki and narrowband UVB phototherapy, was used by 73.3% (n = 88) of the cohort. Significant differences in treatment patterns were observed across vitiligo classifications (*P =* .032) (Table 2), with segmental vitiligo being less frequently treated with dual therapy. Publicly insured patients demonstrated substantially higher denial rates for topical Jaki prescriptions (60% vs 40%; Fisher’s exact *P =* .018). We attempted to investigate an association between FST and VASI scores but were limited by small numbers in FST categories 1, 5, and 6. Across FST categories, the proportion with VASI improvement did not differ significantly (*P* = 0.7) (Table 2).

On multivariable Firth logistic regression adjusting for sex, ethnicity, age, FST, vitiligo classification, dual therapy, and treatment duration, treatment duration remained a strong independent predictor (*P <* .001, adjusted OR [aOR] = 1.11 per month, 95% confidence interval [CI] = 1.04–1.20), with each additional month on treatment increasing the odds of VASI improvement by 11% (Table 4). Ethnicity remained significant, with non-Hispanic patients having 69% lower odds of improvement after adjustment (*P =* 0.034, aOR = 0.307, 95% CI = 0.099–0.912). No other predictors were independently associated. In adjusted logistic regression models with dual therapy as the outcome, segmental vitiligo was independently associated with lower odds of receiving dual therapy (*P* = .025, aOR = 0.114, 95% CI = 0.014–0.756). This finding persisted despite adjustment for demographic and clinical covariates, indicating a different treatment pattern.

**Table 4:** Multivariable Firth Logistic Regression Model Evaluating Predictors of (i) VASI Improvement and (ii) Dual-Therapy Use

Characteristic	OR	95% CI	*P*-Value	OR	95% CI	*P*-Value
	Outcome: VASI Improvement			Outcome: Dual Therapy Use		
Sex (female vs male)						
Male	—	—		—	—	
Female	1.44	0.620–3.44	.4	0.535	0.223–1.24	.14
Ethnicity (non-Hispanic vs Hispanic)						
Hispanic	—	—		—	—	
Non-Hispanic	0.307	0.099–0.912	.034	2.34	0.790–6.86	.12
Age (per y)	1.00	0.980–1.02	.9	0.999	0.978–1.02	>.9
Fitzpatrick Skin Type						
1 (reference)	—	—		—	—	
2	0.570	0.065–7.42	.6	0.406	0.003–4.91	.5
3	1.17	0.144–14.8	.9	0.637	0.005–7.77	.8
4	0.555	0.062–7.33	.6	0.832	0.006–11.1	>.9
5	0.846	0.036–19.4	>.9	1.05	0.005–219	>.9
6	2.31	0.011–119	.7	0.111	0.000–5.78	.3
Dual therapy (yes vs no)						
No	—	—		N/A	N/A	
Yes	1.46	0.511–4.55	.5	N/A	N/A	
Vitiligo classification						
Generalized (reference)	—	—		—	—	
Localized	0.806	0.275–2.31	.7	0.456	0.155–1.27	.13
Acrofacial	2.48	0.804–7.95	.11	0.494	0.145–1.69	.3
Segmental	1.74	0.145–13.3	.6	0.114	0.014–0.756	.025
Treatment duration (per mo)	1.11	1.04–1.20	<.001	0.985	0.932–1.05	.6
VASI improvement (yes vs no)						
No	N/A	N/A		—	—	
Yes	N/A	N/A		1.35	0.511–3.80	.6

Both models adjusted for sex, ethnicity, age, Fitzpatrick Skin Type, vitiligo classification, and treatment duration; the VASI model additionally included dual-therapy status, and the dual-therapy model included VASI improvement. Hispanic ethnicity and longer treatment duration were associated with higher odds of VASI improvement. Segmental vitiligo was associated with lower odds of dual therapy use.

Abbreviations: CI, confidence interval; N/A, not available; VASI, Vitiligo Area Scoring Index.

This study characterizes the real-world vitiligo treatment response to topical Jakis within a diverse Southern California cohort. Compared with the ruxolitinib phase 3 trials, our cohort has greater representation of Asian (34.2%) and Hispanic (16.6%) patients, although representation of Black patients remains limited, reflecting the demographic composition of our Orange County referral population (United States Census Bureau, 2025).

Treatment duration emerged as the stronger independent driver of VASI improvement, with each additional month of treatment increasing the odds of improvement by 11% (aOR = 1.11, 95% CI = 1.04–1.20, *P* < .001). Although this finding is consistent with the well-established time-dependent nature of melanocyte repigmentation—reflecting the gradual recruitment of perifollicular melanocyte stem cells—its confirmation in a real-world diverse cohort is clinically meaningful. It reinforces the importance of counseling patients on the need for sustained treatment adherence before assessing therapeutic response.

Hispanic ethnicity was associated with greater odds of VASI improvement in both bivariate and multivariable analyses (aOR = 0.307 for non-Hispanic vs Hispanic, *P* = .034). However, this finding warrants careful interpretation. Table 1 demonstrates that Hispanic patients in our cohort had a higher proportion of localized vitiligo (45%) than other ethnic groups, and localized disease may be inherently more treatment responsive than generalized or acrofacial subtypes. Although vitiligo classification was included as a covariate in the multivariable model, residual confounding cannot be excluded, given the small sample size. Differential exposures to melanocyte-toxic agents may increase susceptibility to certain subtypes of vitiligo, and future population-based studies with larger sample sizes are necessary to examine these associations.

Segmental vitiligo was independently associated with lower dual therapy use (aOR = 0.114, 95% CI = 0.014–0.756, *P* = .025), consistent with its distinct immunopathogenesis involving somatic mosaicism rather than systemic autoimmunity (Speeckaert et al, 2020). It is important to note that only 5 patients with segmental vitiligo were included in this cohort, precluding any meaningful assessment of treatment response in this subtype. A recent study of 40 patients with segmental vitiligo showed a response to topical 1.5% ruxolitinib cream, suggesting that this subtype may benefit from topical Jakis even without phototherapy and that the lower rate of dual-therapy use observed in this study may not reflect a suboptimal approach (Huang et al, 2026).

Acrofacial vitiligo, involving the hands, feet, and periorificial regions, has historically been considered resistant to topical therapies and phototherapy owing to the relative paucity of hair follicle reservoirs for melanocyte repopulation in acral sites (Speeckaert et al, 2020). It is therefore of interest that the acrofacial subtype did not differ significantly from other subtypes in rates of VASI improvement in our cohort (*P* = 0.2) (Table 2), suggesting that topical Jakis may offer meaningful benefit in this treatment-resistant subtype. However, we also acknowledge an important limitation in interpreting this finding: acrofacial vitiligo as a classification encompasses both facial/periorificial lesions, which tend to respond more readily to treatment given their richer perifollicular melanocyte reservoirs, and true acral lesions of the hands and feet, which are more treatment resistant. Because our analysis does not distinguish between these anatomical sites within the acrofacial category, it is possible that a faster response in facial lesions and a slower response in acral lesions, when combined, produce an aggregate improvement rate that appears comparable to that of other subtypes. We therefore cannot conclude that acral lesions specifically respond as well as other subtypes—this finding may reflect the heterogeneity of the acrofacial classification rather than a true equivalence of response. This study acknowledges that this preliminary observation warrants prospective investigation in larger cohorts with site-specific outcome data.

This study was conducted at an academic dermatology referral center in Orange County, Southern California, a region with a specific demographic composition and insurance landscape, including California’s Medicaid expansion program (Medi-Cal) (United States Census Bureau, 2025). Publicly insured patients in our cohort experienced significantly higher rates of topical Jaki prescription denial (60% vs 40%, *P* = .018). This disparity mirrors patterns previously documented for Jaki access in alopecia areata (Thompson et al, 2020) but has not been previously reported in vitiligo. Given that minority patients are disproportionately represented among publicly insured populations, these insurance barriers may compound the existing clinical gap in treatment access for those who may benefit most.

Strengths of this study include real-world demographic data with a validated VASI scoring system to assess treatment response to a recently Food and Drug Administration–approved therapy. Limitations include the single-center retrospective design and small overall sample size (n = 120), which limits statistical power, particularly for subgroup analyses stratified by vitiligo classification, FST, or race/ethnicity. Small cell sizes across FST 1, 5, and 6 precluded meaningful analysis of FST effects on treatment response. VASI improvement was defined as any decrease in VASI score at final follow-up, a binary classification that does not capture the magnitude or clinical significance of repigmentation; this limitation is acknowledged. In addition, variations in VASI scoring between providers may introduce inconsistencies because significant variations in provider scoring have been previously documented. Standardized VASI scores may lead to increased reproducibility among assessors. Self-reported race and ethnicity are subject to misclassification, and race and ethnicity are socially constructed categories that do not uniformly capture skin pigmentation, ancestry, or biological variation (Harvey et al, 2024; Visscher, 2017). Residual confounding by unmeasured variables—including socioeconomic status, comorbidities, and community-level exposures—cannot be excluded. Black patients and other underrepresented groups were minimally represented in this cohort because of the demographics of the referral population (United States Census Bureau, 2025).

This study highlights real-world disparities in vitiligo care, underscoring the need for improved access to treatment, and provides an initial look into patient variations in treatment response to topical Jakis. Future multicenter studies incorporating additional underrepresented populations, objective pigment measurements such as colorimetry, uniform vitiligo metrics such as VASI scores assessed by the same assessor, and ZIP code–level social determinants of health data could further elucidate community-specific factors that affect treatment response.

## Materials and Methods

### Study design and setting

We conducted a single-institution retrospective cohort study at an academic dermatology referral center in Orange County, Southern California. The study was approved by the institutional review board.

### Patient selection

Patients with an International Classification of Disease, Tenth Revision diagnosis of vitiligo were identified thriugh Epic’s SlicerDicer tool. Inclusion criteria were (i) ≥2 dermatology visits within the study period, (ii) ≥2 recorded VASI scores, (iii) documented insurance information, and (iv) current or prior topical Jaki treatment. Exclusion criteria included fewer than 2 dermatology visits, an absence of recorded VASI scores, or no current or prior topical Jaki treatment. Data were extracted through REDCap on 120 patients evaluated between April 2018 and April 2024.

### Outcomes

Three binary outcomes were examined: (i) VASI improvement, defined as any decrease in VASI score between the first and most recent recorded visit; (ii) dual therapy use, defined as concurrent topical Jaki and narrowband UVB phototherapy at any point during the treatment period; and (iii) topical Jaki treatment approval, defined as initial insurance authorization for the prescribed topical Jaki. Because VASI improvement was defined as any reduction regardless of magnitude, the degree or clinical significance of improvement was not assessed; this limitation is acknowledged as a study limitation.

### Clinical definitions

Vitiligo was classified according to the international consensus framework (Ezzedine et al, 2012). FST was recorded at the clinical assessment. Race and ethnicity were self-reported per institutional intake forms. Race and ethnicity data were collected to characterize the demographic composition of the cohort and to assess demographic disparities in treatment response and access; their collection was not mandated by the funder.

### Missing data

Patients missing VASI scores or key covariates for a given analysis were excluded from that analysis using complete case analysis. Missing data patterns are reflected in the variable totals in Table 2.

### Statistical analysis

Bivariate analyses were conducted using Pearson’s chi-square test, Wilcoxon rank-sum test, or Fisher’s exact test as appropriate. Given the small sample size and outcome prevalence, multivariable Firth penalized-likelihood logistic regression was used for the VASI improvement and dual-therapy models to reduce small-sample bias. Both models were adjusted for sex, ethnicity, age, FST, vitiligo classification, treatment duration, and (where applicable) dual-therapy status or VASI improvement. All analyses were performed using R statistical software, version 4.3.1 (R Foundation for Statistical Computing, Vienna, Austria). A 2-sided *P* < .05 was considered statistically significant.

## Ethics Statement

This was a retrospective institutional review board–approved study (IRB# 3841) at the University of California, Irvine.

## Data Availability Statement

Data related to this article are available upon reasonable request to the corresponding author.

## ORCIDs

Britney Nguyen: http://orcid.org/0009-0004-3812-3572

Hira Rizwi: http://orcid.org/0009-0003-4477-813X

Anastasia Chahine: http://orcid.org/0009-0007-1842-4871

Thabat Dahdoul: http://orcid.org/0009-0004-1915-2482

Jessica Shiu: http://orcid.org/0000-0002-2264-8372

## Conflict of Interest

The authors state no conflicts of interest.
